# Talocalcaneal Tarsal Coalition: A Novel Deltoid-Preserving, Mini-Open Procedure

**DOI:** 10.7759/cureus.94018

**Published:** 2025-10-07

**Authors:** Kenneth Koo, Ahmed Elmahdi, Yara Ibrahim, Edward Gee

**Affiliations:** 1 Trauma and Orthopaedics, Salford Royal NHS Foundation Trust, Manchester, GBR; 2 Trauma and Orthopaedics, Worcestershire Royal Hospital, Worcester, GBR

**Keywords:** foot and ankle surgery, hindfoot valgus, subtalar joint, talocalcaneal coalition, tarsal coalition

## Abstract

Tarsal coalition is an abnormal union of tarsal bones leading to rigid hindfoot valgus and altered biomechanics. Most symptomatic cases involve the talocalcaneal joint. Standard surgical options include resection or arthrodesis, both with limitations. We present a novel deltoid-preserving, mini-open approach designed to restore hindfoot motion while maintaining stability.

A 22-year-old woman presented with severe unilateral hindfoot pain, instability, and valgus deformity. Imaging confirmed a type 3 shingled talocalcaneal coalition involving the sustentaculum tali. Nonoperative options failed, and the patient opted for surgery. A lateral mini-open approach through the sinus tarsi was used to osteotomise the coalition while preserving the superficial deltoid. A small medial approach was used to visualise the sustentaculum tali and protect the medial neurovascular structures. The sustentaculum tali fragment was secured to the calcaneum with a partially threaded screw. Postoperative rehabilitation emphasised subtalar mobility and tibialis posterior strengthening. At 17 weeks, the patient achieved pain-free gait, restoration of the medial arch, symmetrical subtalar motion, and a Manchester-Oxford Foot Questionnaire (MOXFQ) score of 0/64.

Conventional talocalcaneal coalition resections often sacrifice the deltoid ligament and may fail to restore subtalar biomechanics. Arthrodesis relieves pain but increases adjacent joint degeneration risk. Our technique preserves key ligamentous stabilisers, maintains anatomical landmarks, and restores subtalar motion. Literature suggests that long-term issues after standard resections include persistent flatfoot and abnormal mechanics, highlighting the potential benefit of this approach.

This novel deltoid-preserving, mini-open procedure may provide adult patients with an alternative to arthrodesis or traditional resection, offering pain relief, preservation of hindfoot motion, and improved biomechanics.

## Introduction

Tarsal coalition is an abnormal connection between two or more tarsal bones resulting from the failure of embryonic segmentation. It may be congenital or acquired, with congenital variants being more common. Congenital coalitions typically begin as fibrous unions (syndesmoses) and may progress to cartilaginous (synchondroses) or osseous (synostoses) fusions during adolescence [1]. Embryologically, the condition results from the incomplete differentiation of primitive mesenchyme into cartilage, leaving persistent non-physiological connections [2].

The prevalence of tarsal coalition in the general population is estimated at approximately 2%, though cadaveric studies have reported rates as high as 13% [3]. The most frequently involved joints are the talocalcaneal and calcaneonavicular [4]. Among these, 45% occur at the middle facet of the subtalar joint [4]. While many individuals remain asymptomatic, symptomatic patients often present with rigid planovalgus deformity, activity-related hindfoot pain, recurrent ankle sprains, or functional instability.

The subtalar joint plays a critical role in lower limb biomechanics, allowing the hindfoot to move from valgus at heel strike to varus at toe-off. This motion unlocks and locks the Chopart joints, enabling the foot to act alternately as a flexible shock absorber and a rigid lever for propulsion [5]. In the presence of a coalition, subtalar inversion and eversion are restricted, altering load transfer across the foot. The resulting abnormal stresses predispose to midfoot arthritis, ligamentous strain, peroneal spasm, and recurrent ankle injuries [6].

Radiographic diagnosis is aided by classical signs such as the talar beak, narrowing of the posterior facet, and the "C-sign", while computed tomography (CT) and magnetic resonance imaging (MRI) provide superior characterisation and are essential for surgical planning [7].

Although nonoperative management, including orthoses, physiotherapy, and casting, may provide symptom relief, surgical intervention is often required in persistent cases. Current surgical options include coalition resection, deformity correction, or subtalar arthrodesis, each with distinct advantages and limitations [4,8]. In adults, arthrodesis is frequently selected due to concerns regarding resection outcomes, but this sacrifices subtalar motion and may accelerate adjacent joint degeneration [5,9].

More recently, minimally invasive techniques such as arthroscopy and endoscopy have been introduced as alternatives to open surgery. These approaches allow coalition resection or subtalar fusion to be performed with less soft tissue disruption, reduced morbidity, and potentially faster recovery. Arthroscopic resection has shown promising results in selected patients, particularly in talocalcaneal coalitions, with improvements in pain and function comparable to open techniques. However, the procedures are technically demanding and require specialised expertise, and the long-term outcomes remain less well-established compared with conventional open approaches.

In light of these challenges, there is a growing need for techniques that relieve pain, restore subtalar mobility, and preserve the function of stabilising soft tissues. This study presents a novel deltoid-preserving, mini-open procedure for the management of symptomatic middle facet talocalcaneal coalition in an adult patient.

## Case presentation

History

A 22-year-old fit and active woman presented with a unilateral (left) painful hindfoot. Pain was described as deep, beneath both malleoli but worse medially, and graded as severe during or after activity. She was unable to run, could not wear heeled footwear, and reported difficulty standing for prolonged periods at work. She also noted instability, describing episodes of the ankle "giving way", and had sustained a previous ankle sprain as an adolescent. She had no relevant past medical history and no family history of foot deformities. A Manchester-Oxford Foot Questionnaire (MOXFQ) score was recorded as 36/64.

Examination

The patient was exquisitely tender over the sustentaculum tali, with a valgus heel position and no detectable subtalar motion. She could perform a double heel raise with discomfort but without correction of hindfoot valgus and was unable to perform a single heel raise. Midfoot and forefoot examinations were normal.

Imaging

A weight-bearing lateral radiograph revealed the classical "C-sign", a pathognomonic feature of talocalcaneal coalition, but no talar beaking. Meary's line was preserved.

**Figure 1:**
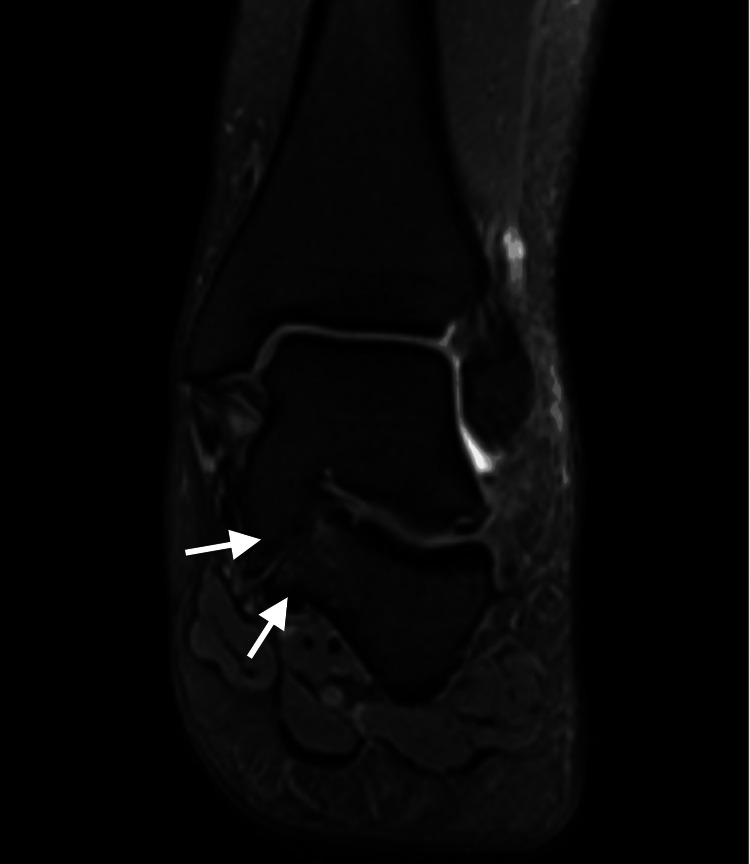
Coronal MRI demonstrating a type 3 shingled talocalcaneal coalition with fusion of the sustentaculum tali to the talus MRI: magnetic resonance imaging

MRI demonstrated a type 3 shingled talocalcaneal coalition, in which the sustentaculum tali of the calcaneus was fused to the body of the talus, with a non-osseous coalition between the base of the sustentaculum tali and the calcaneus (Figure 1). The entire middle facet was included in the osseous coalition, while the posterior and anterior facets were preserved but showed chondral thinning.

After discussion of operative and nonoperative treatment options, the patient elected for surgery. Published studies have shown good function and pain relief up to one year after the resection of middle facet tarsal coalitions [3]. Our aim was to restore hindfoot motion and limit further subtalar degeneration.

Surgical technique

**Figure 2:**
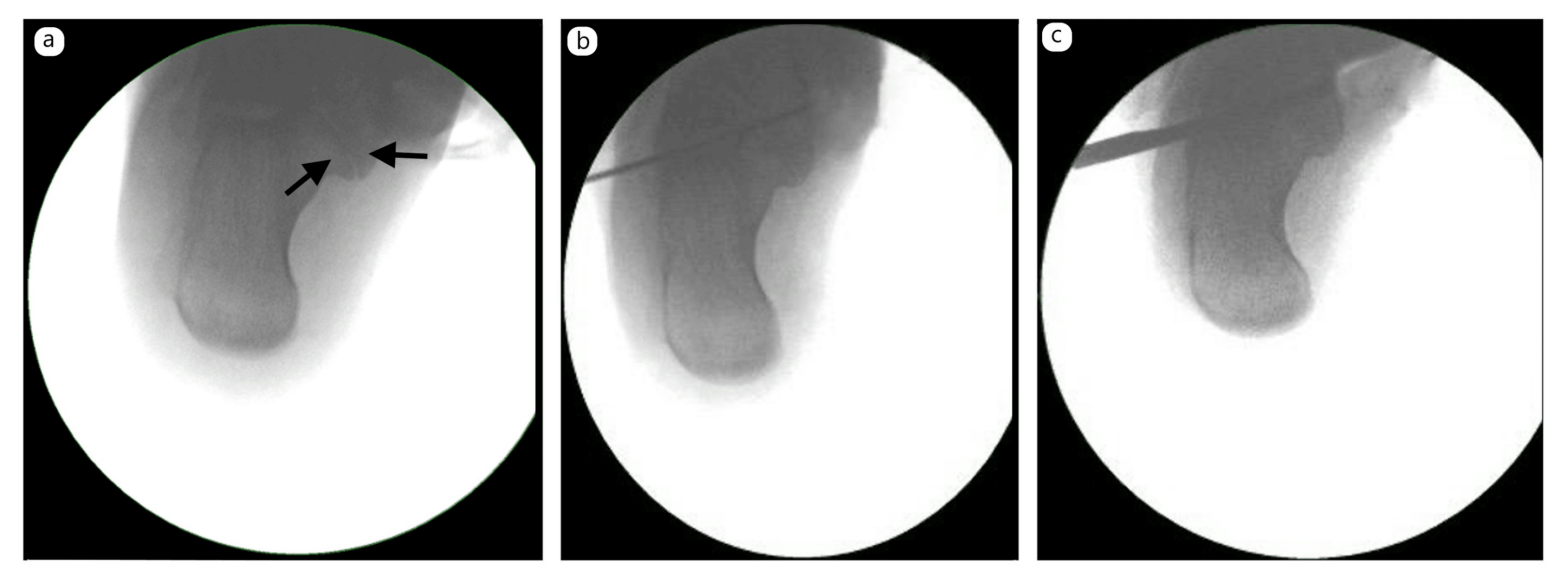
Intraoperative fluoroscopic images: (a) visualisation of the coalition; (b) placement of two 2-mm K-wires across the sinus tarsi to define the osteotomy plane; and (c) use of an 8-mm Lambotte osteotome guided by the K-wires

Initial attempts to pass a small joint arthroscope into the subtalar joint were unsuccessful. A 1-cm lateral incision was therefore used to access the sinus tarsi. A 5-cm medial incision was made to expose the sustentaculum tali while protecting medial neurovascular structures, with careful dissection of the tibialis posterior and flexor digitorum longus tendon sheaths. Two 2-mm smooth K-wires were passed laterally to medially under fluoroscopy to define the osteotomy plane at the middle facet and to perforate the medial cortex without violating the superficial deltoid ligament (Figure 2a-2b).

An 8-mm Lambotte osteotome was passed laterally to medially, completing the osteotomy without injuring the superficial deltoid (Figure 2c). Arthroscopy and shaver debridement were performed to clear debris and smooth the osteotomy bed. The posterior and anterior facets were preserved with good articular surfaces, and subtalar mobility was restored.

**Figure 3:**
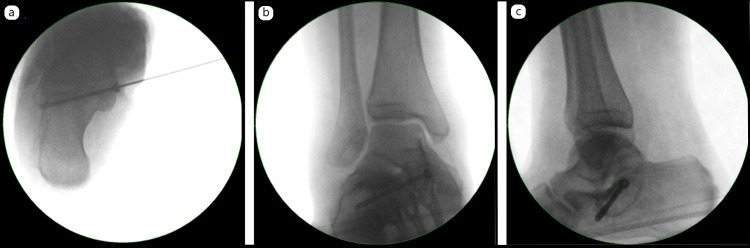
Intraoperative fluoroscopic images: (a) fixation of the sustentaculum tali fragment with a cannulated screw; (b) anteroposterior view; and (c) lateral view showing stable fixation

A further 2-mm K-wire was advanced across the sinus tarsi through the superficial deltoid ligament to confirm the subtalar plane. A guidewire was then passed through the sustentaculum tali into the fibrous coalition, repeatedly perforating to stimulate bleeding bone. A 4-mm partially threaded titanium cannulated screw was inserted to compress the sustentacular fragment to the calcaneus (Figure 3).

Passive intraoperative testing confirmed satisfactory inversion and eversion at the subtalar joint, with stable fixation of the sustentaculum tali. The medial tendon sheaths were repaired, and the patient was placed in a removable walking boot.

Postoperative course

Postoperatively, the patient remained non-weight-bearing for the first two weeks, during which early sagittal range of motion exercises were initiated. Between two and four weeks, touch weight-bearing was permitted in a boot, and active subtalar motion was encouraged to maintain joint mobility. From four to six weeks, the patient progressed to partial weight-bearing of approximately 50%, continuing protected mobilisation in the boot. By six to eight weeks, full weight-bearing was allowed as tolerated, with a gradual return to normal function.

Clinical results

At 10 weeks postoperatively, the patient reported resolution of deep ankle pain and complete wound healing but described medial arch discomfort and calf fatigue with prolonged activity. Examination revealed physiological pes planus and tibialis posterior weakness. Radiographs confirmed union of the sustentaculum tali fragment to the calcaneus. She commenced targeted physiotherapy for tibialis posterior strengthening.

**Figure 4:**
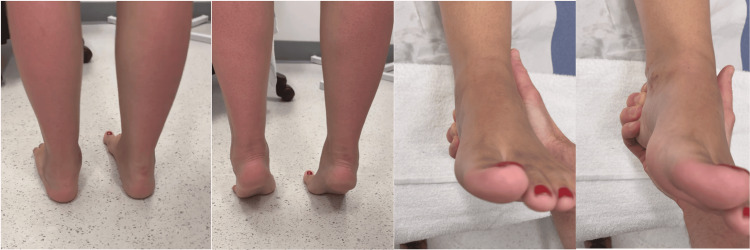
Clinical images at 17 weeks postoperatively showing the restoration of range of motion and medial arch reconstitution

At 17 weeks, gait had normalised with the restoration of the medial longitudinal arch. Subtalar range of motion was symmetrical, and the tiptoe stance demonstrated inversion of the heel. The patient was pain-free and ambulating in normal footwear. The MOXFQ score had improved to 0/64 (Figure 4).

## Discussion

Arthrodesis for talocalcaneal coalitions is an option for middle facet coalitions when more than 50% of the subtalar joint is affected by coalition or when the remaining articular surface shows signs of degeneration [4,8,10]. The aim of the arthrodesis is to correct the hindfoot deformity so that less valgus stress and stretching of the ligamentous and tendinous structures result. Correction of the hindfoot deformity was noted to improve the range of motion in dorsiflexion and plantarflexion in one case report [8].

Arthrodesis can provide predictable improvement in pain, but it does not allow for the normal biomechanics of the subtalar joint [5]. Fusion of the subtalar joint means that the midfoot joints will compensate, seen with an increase in external rotation of the tibia on normal loading of cadaveric specimens [5] and radiological evidence of osteoarthritis in adjacent joints on long-term follow-up studies [9]. In the young and active population, arthritis in the neighbouring joints will be more likely to occur following arthrodesis due to the compensatory movements in these joints. Table 1 shows the classification of talocalcaneal tarsal coalition based on axial cross-sectional images.

**Table 1:** Classification of talocalcaneal tarsal coalition based on axial cross-sectional images

	Classification	Description
Type 1	Linear	Parallel to the direction of the subtalar joint
Type 2	Linear with posterior hook	Linear anteriorly in the subtalar joint but curves into a posterior hook medially, over and behind the sustentaculum tali
Type 3	Shingled	Related to a hypoplastic sustentaculum tali with an overlap of the talar portion over the calcaneum
Type 4	Complete osseous	A complete ossification of the sustentaculum tali and calcaneal bridge
Type 5	Posterior	Small coalitions lying directly under the posterior tibial artery

Resection of the coalition is another surgical option for talocalcaneal coalitions and is often performed through a medial approach from the tip of the medial malleolus to the sustentaculum tali. Once the coalition is exposed and osteotomised, the remaining space is interposed with fat tissue or bone wax. Variations of this technique have also been performed with the interposition of the long flexor tendons or the extensor digitorum brevis.

Triple arthrodesis has also been reported in the literature, demonstrating reliable pain relief but similarly associated with long-term altered biomechanics and degenerative changes [11].

The advantage of resection over arthrodesis is the restoration of a flexible hindfoot that allows more normal midfoot mechanics. Wilde et al. were one of the first to demonstrate painless subtalar motion at 12-month follow-up following the resection of the talocalcaneal coalition [12]. This group also highlighted that indications for resection included those with middle facet coalitions with an involvement of less than 50% of the subtalar joint on CT scan, a hindfoot valgus of less than 16°, minimal joint degeneration of the posterior talocalcaneal joint, and the absence of lateral talar process impingement on the calcaneum to ensure postoperative symptomatic relief [12]. Poor results have been reported when the above criteria aren't met, with secondary degeneration and pain in the remainder of the subtalar joint due to increased contact stresses and motion in an already degenerate joint [3].

With the use of the validated American Orthopaedic Foot and Ankle Society (AOFAS) Ankle-Hindfoot score, several studies have highlighted pain relief following coalition resection in patients following Wilde et al.'s indications, up to 25 years post-surgery [10,13].

Contrary to Wilde et al., Luhmann and Schoenecker showed that some patients with coalitions greater than 50% involvement of the subtalar joint had fared better than preoperatively in terms of pain relief [14]. Khoshbin et al. also demonstrated favourable long-term outcomes following resections of coalitions with greater than 50% involvement and a hindfoot valgus greater than 16° [13]. In this study, two validated patient-reported outcome measures (PROMs) were used to compare individuals with a hindfoot valgus angle greater than 16° to those with a valgus angle less than 16°. The results demonstrated no significant difference in outcomes between the two groups, regardless of the size of the coalition [13]. The study suggests that resection is a viable option prior to the consideration of arthrodesis. It is noted in all these studies that patients were adolescents and all presented with planovalgus on weight bearing at follow-up [10,12,14]. This finding has been consistently reported in subsequent studies, highlighted by a worsening of the hindfoot valgus following resection [15].

One study compared the activity levels of patients with talocalcaneal coalitions who opted for excision and those who continued nonoperative treatment [16]. Those opting for resection were able to return to sporting activity, whereas those who were treated nonoperatively had stopped all sporting activity [16]. In particular, those who had a resection during adulthood were able to regain their desired activity levels, with syndesmotic coalitions returning to high levels of sporting activity [16]. It is noted that these athletes had worn orthotics post-resection.

Resection can also be performed with hindfoot deformity corrections, where some surgeons have shown symptom control and a reorientation of the Chopart joint to act as a "pseudo-subtalar" joint for inversion and eversion [4,14,17]. In patients with a hindfoot valgus greater than 21°, Luhmann and Schoenecker recommended a medialising calcaneal osteotomy or a lateral column lengthening calcaneal osteotomy (Evan's osteotomy) as an alternative to arthrodesis [14]. This was also a view shared by Mosca and Bevan, who performed a lateral column calcaneal lengthening osteotomy and lengthening of the gastrocnemius (Strayer's procedure) with and without tarsal coalition resection [4]. What can be concluded from these studies is that hindfoot deformity correction is reserved for those with severe hindfoot valgus and has shown significant improvement in pain relief.

Resection of the coalition does not address the chronically deformed soft tissues, and the pes planovalgus may continue after resection [3,15].

The normal gliding and rotatory subtalar joint kinematics following resection has not been shown to be restored despite significant clinical improvement in pain [18]. On observation of dynamic gait in patients post-resection, these patients exhibit the same inversion/eversion subtalar restrictions as pre-resection, despite a passively mobile hindfoot [18]. Abnormal findings were also found on electromyography of the peroneal muscles and the gastro-soleus complex, with increased spasticity in post-resection patients on walking [19]. Moreover, increased plantar pressures in the midfoot, with a consistently reduced loading of the fifth metatarsal head in patients post-resection, suggest that the mid- and forefoot rotation is still present, causing a reduced medial arch [19]. These abnormal mechanics would lead to increased load contact pressures and torque on adjacent joints and potentially secondary osteoarthritis [16].

One recent study revealed radiographic evidence of subtalar joint osteoarthritis at five-year follow-up in patients following talocalcaneal coalition resections [20].

Outcomes following surgical treatment in adult tarsal coalitions are less known due to the paucity of studies in this population. Recent review articles currently advocate for the restoration of hindfoot motion in the adult population [10]. With this in mind, we propose a novel technique to restore the hindfoot motion and the biomechanics of the foot for the treatment of middle facet talocalcaneal coalitions in an adult.

## Conclusions

This novel surgical technique aims to restore the anatomical structures of the ankle, converting a rigid planovalgus into a flexible planovalgus. It offers an additional option for the management of tarsal coalitions diagnosed in adulthood. Furthermore, correction of the biomechanics of the flexible planovalgus can be optimised by targeting the posterior tibialis with eccentric and concentric exercises, supplemented by orthotics.

In the described techniques of talocalcaneal resections, all approaches were made medially. With a medial approach, the medial soft tissues are sacrificed and allowed to heal with scar tissue, namely, the superficial deltoid. The senior author describes a technique of resection of the coalition from a lateral approach, thus maintaining the integrity of the superficial deltoid ligament and flexor retinaculum.

We believe that with this technique, combined with a structured physiotherapy protocol and orthotic support, restoration of more normal subtalar biomechanics can be achieved.
